# Aberrant Functional Connectivity and Structural Atrophy in Subcortical Vascular Cognitive Impairment: Relationship with Cognitive Impairments

**DOI:** 10.3389/fnagi.2016.00014

**Published:** 2016-02-02

**Authors:** Xia Zhou, Xiaopeng Hu, Chao Zhang, Haibao Wang, Xiaoqun Zhu, Liyan Xu, Zhongwu Sun, Yongqiang Yu

**Affiliations:** ^1^Department of Neurology, The First Affiliated Hospital of Anhui Medical University, Anhui, China; ^2^Department of Radiology, The First Affiliated Hospital of Anhui Medical University, Anhui, China

**Keywords:** resting-state fMRI, functional connectivity, gray matter atrophy, thalamus, MPFC, vascular cognition impairment

## Abstract

Abnormal structures in the cortical and subcortical regions have been identified in subcortical vascular cognition impairment (SVCI). However, little is known about the functional alterations in SVCI, and no study refers to the functional connectivity in the prefrontal and subcortical regions in this context. The medial prefrontal cortex (MPFC) is an important region of the executive network and default mode network, and the subcortical thalamus plays vital roles in mediating or modulating these two networks. To investigate both thalamus- and MPFC-related functional connectivity as well as its relationship with cognition in SVCI, 32 SVCI patients and 23 control individuals were administered neuropsychological assessments. They also underwent structural and functional magnetic resonance imaging scans. Voxel-based morphometry and functional connectivity analysis were performed to detect gray matter (GM) atrophy and to characterize the functional alterations in the thalamus and the MPFC. For structural data, we observed that GM atrophy was distributed in both cortical regions and subcortical areas. For functional data, we observed that the thalamus functional connectivity in SVCI was significantly decreased in several cortical regions [i.e., the orbitofrontal lobe (OFL)], which are mainly involved in executive function and memory function. However, connectivity was increased in several frontal regions (i.e., the inferior frontal gyrus), which may be induced by the compensatory recruitment of the decreased functional connectivity. The MPFC functional connectivity was also decreased in executive- and memory-related regions (i.e., the anterior cingulate cortex) along with a motor region (i.e., the supplementary motor area). In addition, the cognitive performance was closely correlated with functional connectivity between the left thalamus and the left OFL in SVCI. The present study, thus, provides evidence for an association between structural and functional alterations, and sheds light on the underlying neural mechanisms of executive dysfunction in SVCI.

## Introduction

Vascular dementia (VD) is currently thought to be the second most common type of dementia after Alzheimer’s disease (AD). Vascular cognitive impairment (VCI) is rapidly drawing attention because of its high prevalence and potential reversibility as well as its symptoms, which include all levels of cognitive loss, from mild deficits to VD (O’Brien et al., 2003). Subcortical VCI (SVCI) refers to a homogeneous form of VCI, characterized by extensive white matter hyperintensities (WMH) and multiple lacunar infarctions on magnetic resonance imaging (MRI) (de Mendonca et al., 2005). The most striking cognitive impairment in SVCI is executive and attention deficits (Erkinjuntti, 2003). Furthermore, SVCI has been proposed to be associated with an increased risk of dementia, stroke, and even death (Debette et al., 2010). Although the definite pathophysiological mechanism of SVCI is not clear, converging evidence indicates that frontal-subcortical loops may subserve the functions of executive/attention (Bonelli and Cummings, 2007), and that disturbance of these loops may disrupt information processing, leading to the hallmark symptoms of SVCI.

Over the past few years, accumulating neuroimaging studies have greatly advanced our understanding of the pathogenesis of SVCI (Seo et al., 2010; Sun et al., 2011; Yi et al., 2012; Thong et al., 2014; Lin et al., 2015). The results of structural MRI studies have shown that both gray matter (GM) and white matter are aberrant in SVCI (Seo et al., 2010; Yi et al., 2012; Thong et al., 2014; Lin et al., 2015). A voxel-based morphometry (VBM) study (Thong et al., 2014) demonstrated that patients with mild SVCI had widespread volume shrinkage, especially in the regions of the frontal cortex and subcortical areas. Specifically, in Seo’s study (Seo et al., 2010), frontal cortical thinning was closely associated with executive dysfunction in subcortical vascular mild cognitive impairment (SVMCI) patients. A recent study (Lin et al., 2015) using diffusion tensor imaging (DTI) found that SVCI patients exhibited reduced fractional anisotropy (FA) in the whole brain, which added to the evidence of the white matter tract damage in SVCI.

Moreover, functional MRI (fMRI), which is based on low-frequency blood oxygen-level dependent (BOLD) fluctuations, provides us a promising viewpoint to explore the changes in spontaneous neural activity in SVCI. In 2011, Sun et al. (2011) first revealed abnormal functional connectivity between the posterior cingulate cortex (PCC) and the frontal and temporal regions using resting-state fMRI in patients with VCI, no dementia (VCIND), a clinical state with a vascular etiology similar to that of SVCI. Subsequently, Yi et al. (2012) observed significant functional alterations of low-frequency oscillation (LFO) amplitudes in the default mode network (DMN) in SVMCI. Notably, the LFO amplitudes in the medial prefrontal cortex (MPFC) were decreased, whereas those in the PCC were increased. The DMN is a distributed network of brain regions, which includes the MPFC, the PCC, the anterior cingulate cortex (ACC), and so on, and it is known to be active at baseline but inactive during tasks, thereby subserving a succession of cognition, including episodic memory processing and self-referential processing (Greicius et al., 2003; Sun et al., 2011). The MPFC is the anterior hub of the DMN and has even been deemed as a control center in executive network, contributing to numerous cognitive activities (Unschuld et al., 2013; Riga et al., 2014). The PCC is the node with the highest metabolic activity in DMN (Miao et al., 2011). Structural and functional connections have been established between the MPFC and the PCC and other regions in the brain (Khalsa et al., 2014), which make both the MPFC and the PCC especially interesting and meaningful in the study of functional connectivity in SVCI.

Previous studies have focused mainly on the cortical region; less attention has been devoted to the potential role of subcortical nuclei. According to previous studies (Schmahmann, 2003; Zhang et al., 2008), the thalamus, with its dense cortical and subcortical connections, is a vital subcortical region that supports cognitive functions that are known to be reduced in SVCI, including executive/attention functions and memory, among others. Studies have provided evidence of functional metabolic and structural abnormalities in the thalamus in SVCI patients. A fluoro-2-deoxy-d-glucose-positron emission tomography (FDG-PET) study (Seo et al., 2009) has demonstrated prominent hypometabolic involvement of the subcortical areas, especially the thalamus instead of the frontal cortices. Shape analyses (Yi et al., 2012) of brain structures have also shown a significantly reduced volume of the thalamus in SVCI. Moreover, the thalamus plays vital roles in mediating or modulating the above DMN and executive networks via its dense cortical and subcortical connections (Yuan et al., 2015). However, less is known regarding functional connectivity alteration in the thalamus and its relationship with cognition in SVCI patients, despite the central role of the thalamus in the function of the cerebral cortex.

Therefore, in the present study, we sought to compute functional brain connectivity in both the cortical and the subcortical regions and to evaluate the effect of functional alterations on cognitive impairment in patients with SVCI using resting-state fMRI. We hypothesized that resting-state thalamus and MPFC functional connectivity with all other brain regions would be disrupted in SVCI patients.

## Materials and Methods

### Participants

A total of 55 right-handed participants, including 32 SVCI patients and 23 healthy controls from the First Affiliated Hospital of Anhui Medical University, participated in this study. Each subject provided written informed consent to participate in the study. Because severe cognitive disability can make it impossible to obtain truly informed consent, we also obtained written informed consent from the patients’ caretakers. This study was approved by the Institutional Review Board of the First Affiliated Hospital of Anhui Medical University Subcommittee on Human Studies.

The patients with SVCI met the following inclusion criteria (Park et al., 2014): (1) subjective cognitive complaints by the patient or his/her caregiver, including executive dysfunction, processing speed deficits, or memory deficits; (2) objective cognitive impairments in any aspect, including impairments in memory, language, executive function, attention, or visuospatial ability, based on neuropsychological testing; (3) significant ischemia, including significant WMH on MRI scans (Fazekas score 2–3) or lacunar infarctions; (4) evident subcortical vascular signs of dysarthria, sensory deficits, gait disorder, urgent urination, motor slowness, dysarthria, or lower facial weakness detected by neurological examination or reported by the patient; (5) age ≥50; and (6) any vascular risk factors, including diabetes, hypertension, hypercholesterolemia, smoking, or a positive family history of stroke. The 23 healthy controls were recruited either from the spouses of the patients or via advertisement. The healthy control subjects had no significant cognitive complaints or neuropsychiatric diseases and were not using any psychoactive medicines. Additionally, all were required to undergo the mini-mental state examination (MMSE) and to receive a score of 26 or higher based on an evaluation designed for subjects with at least 5 years of education.

The exclusion criteria were: (1) a history of alcoholism, head injury, Parkinson’s disease, epilepsy, multiple sclerosis, major depression or other neurological or psychiatric illness, or major medical illness; (2) early onset of memory deficit and progressive worsening of memory and other cognitive cortical functions; (3) a history of intracranial hemorrhage; (4) any cortical infarct or an infarct of ≥15 mm in diameter at any location upon routine MRI; and (5) severe vision or hearing loss or the presence of dentures or metallic stent *in vivo*.

All subjects completed a neuropsychological battery, including the MMSE, the clinical dementia rating (CDR), the Cambridge Cognitive Examination-Chinese version (CAMCOG-C), the Montreal Cognitive Assessment (MoCA), the activities of daily living (ADL) scale, a Stroop test, and the Global Deterioration Scale (GDS), for the purpose of evaluating the global cognition, memory function, attention, visuospatial skills, processing speed, executive function, and emotion, respectively. The administration of the battery took between two and two and a half hours.

### MRI Data Acquisition

Magnetic resonance imaging for all participants was performed on a 3.0-T GE Signa HDxt MRI scanner (GE, Milwaukee, WI, USA). A high-resolution T1-weighted anatomical image was collected (TR = 9.5 ms; TE = 3.9 ms; TI = 450 ms; flip angle = 20°; field of view = 256 mm; matrix size = 512 × 512). After structural MRI scans, resting-state functional images, compiled from 240 contiguous echo planar images, were acquired using an echoplanar imaging (EPI) sequence with the following parameters: TR = 2 s, TE = 30 ms, FOV = 240 mm, flip angle = 80°, matrix size = 64 × 64, thickness = 4 mm, and gap = 0.6 mm. During the resting scan, subjects were asked to lie quietly and still, with their eyes closed, and to clear their heads of all thoughts. All resting-state fMRI data preprocessing was conducted using statistical parametric mapping (SPM8)^1^ and data processing assistant for resting-state fMRI (DPARSF) (Chao-Gan and Yu-Feng, 2010).

### Data Analysis

Functional images were preprocessed and analyzed with the functional connectivity toolbox for correlated and anticorrelated brain networks (CONN) on the MATLAB platform (Whitfield-Gabrieli and Nieto-Castanon, 2012). The first 10 volumes of the scanning session were discarded to allow for T1 equilibration effects. The preprocessing steps included conventional slice timing correction, realignment, coregistration, normalization, and spatial smoothing with an 8-mm Gaussian kernel of full width at half maximum. Temporal processing was performed using a component-based noise correction method (Comp Cor) (Whitfield-Gabrieli and Nieto-Castanon, 2012). The removed temporal confounding factors included motion parameters, cardiac, respiratory, and other physiological noise, and a global BOLD signal; these were removed by identifying significant principal components derived from regions of interest (ROIS). The BOLD signals from the white matter and cerebrospinal fluid (CSF) masks were analyzed as additional covariates. Next, a temporal band-pass filter (0.01 < *f* < 0.08 Hz) was employed to remove low-frequency drift and high-frequency noise. Seed-to-voxel connectivity was then measured using the CONN toolbox. For this study, we focused on the thalamus and the MPFC because of their vital roles in functional convey in SVCI. The seed regions were defined by the software WFU PickAtlas Tool Version 2.4^2^ (Ledberg et al., 1998). Entire BOLD time courses were extracted from these seed regions and from all other voxels. In addition, Pearson’s correlation coefficients were calculated between the time courses of the seed regions and those of all other voxels across the brain. Fisher-*z*-transformation was applied to convert the resulting correlation coefficients. Second-level general linear model analyses were performed on the *z*-scores. The threshold for significance was *p* < 0.05. Family wise error (FWE) correction with clustering was performed using a cluster-forming threshold. The results of the exploratory analyses were considered significant if clusters survived FWE correction at *p* < 0.05.

### Voxel-Based Morphometry

Structural MRI data processing was performed using VBM and the diffeomorphic anatomical registration using exponentiated Lie algebra (DARTEL) registration method (Ashburner, 2007). Anatomical images were first segmented into GM, white matter, and CSF as well as into three extra-cerebral tissue classes. GM maps were then normalized to the GM population-specific template generated from the complete image set using DARTEL. Spatially normalized images were then modulated by the Jacobian determinants derived from the spatial normalization, and then smoothed with an 8-mm full width at half maximum kernel. Next, the automated anatomical labeling (AAL) template was resampled in the DARTEL space. Global GM volumes for each subject were calculated as the mean value of all the voxels within all regions.

### Statistical Analysis

The data are presented as the mean ± SD for normally distributed variables (all continuous data except CDR data) and as the median (minimum–maximum) for the skew-distributed continuous variables (CDR data). The Pearson chi-square test method for categorical data, a two-tailed *t*-test for normally distributed continuous data, and the Mann–Whitney U test for the skew-distributed parameters were performed at a threshold with *p* < 0.05. Pearson’s correlation analysis was performed to explore the relationships between functional connectivity strength (*z*-scores) and cognitive performances of SVCI patients. False discovery rate (FDR) was performed to correct the multiple comparisons for the *p*-value. Statistical significance was taken at *p* < 0.05.

## Results

### Clinical Characteristics

Table 1 shows the subjects’ demographic and clinical characteristics. No significant differences were found between the groups in terms of age, sex, or years of education. However, SVCI patients exhibited significantly lower global cognitive scores, as indicated by the MMSE, CAMCOG-C, ADL scale, and CDR. Higher ADL scores in the SVCI group indicated dysfunction in daily living compared with the controls. Regarding the subtypes of cognition, SVCI patients took much more time in all types of Stroop performance and had lower scores for the subscales of the CAMCOG-C (praxis, attention), which indicated the deficits in executive/attention function in these patients.

**Table 1:** **Demographic and neuropsychological data**.

		SVCI (*n* **=** 32)	Controls (*n* **=** 23)	*p*-value
Gender (male%)		43.80	60.90	0.277^a^
Age (years)		70.09 ± 8.26	68.87 ± 7.05	0.557^b^
Years of education		8.47 ± 3.16	10.09 ± 2.98	0.059^b^
MMSE		23.78 ± 2.66	27.96 ± 0.98	<0.001^b^
CAMCOG-C		76.78 ± 9.26	92.83 ± 4.63	<0.001^b^
	Subscales of CAMCOG-C			
Orientation		8.75 ± 1.30	9.87 ± 0.34	<0.001^b^
Language		22.94 ± 2.11	26.96 ± 1.80	<0.001^b^
Memory		18.12 ± 4.30	22.09 ± 2.33	<0.001^b^
Attention		4.94 ± 1.52	6.48 ± 0.79	0.023^b^
Praxis		8.75 ± 2.37	11.04 ± 0.98	<0.001^b^
Calculation		1.84 ± 0.37	2.00 ± 0.00	<0.001^b^
Abstraction		4.84 ± 1.63	6.52 ± 0.85	<0.001^b^
Perception		6.59 ± 1.43	7.87 ± 1.39	0.002^b^
ADL scale		25.47 ± 7.42	20.22 ± 0.70	<0.001^b^
CDR		0.5 (0.5–2.0)	0	<0.001^c^
Stroop (dot)		38.38 ± 13.82	24.04 ± 8.93	<0.001^b^
Stroop (characters)		47.13 ± 12.25	30.61 ± 9.08	<0.001^b^
Stroop (color)		68.94 ± 19.90	46.26 ± 10.86	<0.001^b^

*The data are presented as the mean ± SD MMSE, mini-mental state examination; CAMCOG-C, Cambridge Cognitive Examination-Chinese version; ADL, activities of daily living; GDS, global deterioration scale; CDR, clinical dementia rating*.

*^a^Two-tailed Pearson chi-square test*.

*^b^Two-sample two-tailed *t*-test*.

*^c^Mann–Whitney *U* test*.

### Between-Group Functional Connectivity Difference

#### Group Difference in the Left Thalamus and the Right Thalamus Connectivity

**Figure 1:**
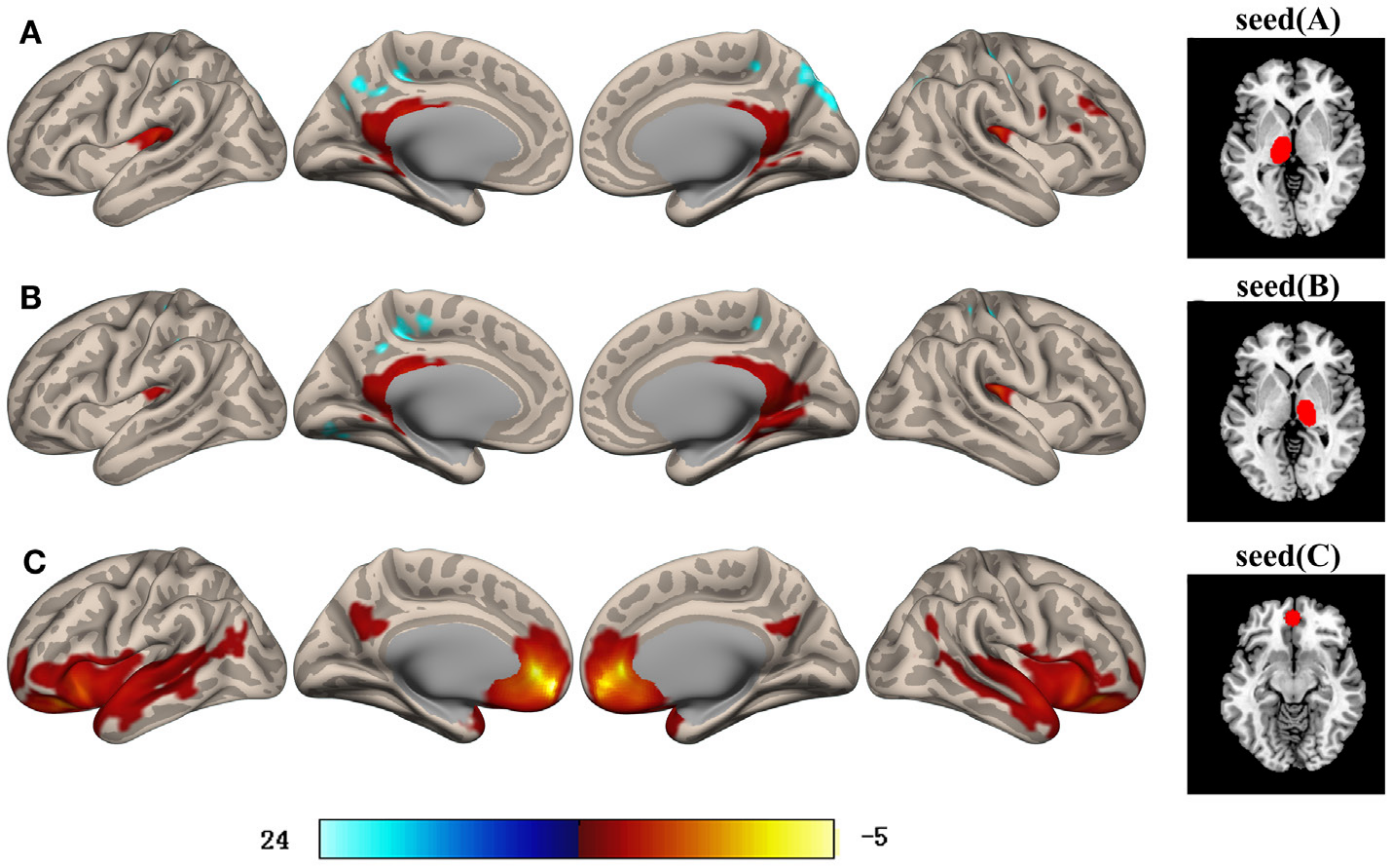
**Brain areas with significant differences in their functional connectivity with the left thalamus (A), right thalamus (B), and MPFC (C) between SVCI patients and the controls (*p* < 0.05, FWE-corrected)**. Orange/hot and blue represent decreased and increased functional connectivity, respectively. The color bar indicates the *t*-value. Intriguingly, the MFG and IFG showed increased connectivity with the bilateral thalamus in SVCI group. Details of the clusters are shown in Table 2 (FWE-corrected, threshold of *p* = 0.05). MFG, middle frontal gyrus; IFG, inferior frontal gyrus.

The SVCI group exhibited hypoconnectivity between the bilateral thalamus and the orbitofrontal lobe (OFL), fusiform gyrus (FFG), insula (INS), and middle temporal gyrus (MTG) when compared with the controls. The SVCI group also showed significantly decreased functional connectivity between the left thalamus and the right superior temporal gyrus (STG.R), left superior frontal gyrus (SFG.L), and putamen (PUT.L). For the right thalamus, decreased functional connectivity with the left inferior temporal gyrus (ITG.L) was found. By contrast, increased connectivity between the bilateral thalamus and the regions of the right inferior frontal gyrus (IFG.R) and between the bilateral thalamus and the right middle frontal gyrus (MFG.R) was observed (Table 2; Figure 1).

**Table 2:** **Regions showing functional connectivity differences in the regions of interest (ROIS) between the SVCI patients and the control subjects (*p* < 0.05, 40 voxels, corrected for FWE)**.

ROI	Brain regions	Peak voxel coordinate	Cluster size (KE)	*z*-scores	*t-*scores	Cluster significance (FDR-corrected, threshold of *p* **=** 0.05)	Cluster significance (FWE-corrected, threshold of *p* **=** 0.05)
Left thalamus	MTG.R, STG.R	10 36 42	3797	−0.1	−7.73	*p* < 0.001	*p* < 0.001
	MFG.L, OFL.L SFG.L, PUT.L	22 28 36	1238	−0.1	−7.73	*p* < 0.001	*p* < 0.001
	MFG.R, IFG.R	48 44 24	845	0.1	4.85	*p* < 0.001	*p* < 0.001
	OFL.R, INS.R	28 22 08	553	−0.1	−4.93	*p* < 0.001	*p* < 0.001
	MTG.R, STG.R, FFG.R	64 02 12	193	−0.08	−4.71	0.0023	*p* < 0.001
Right thalamus	MTG.L, ITG.L, FFG.L	16 70 12	11,996	−0.1	−10.62	*p* < 0.001	*p* < 0.001
	IFG.R, MFG.R	32 60 02	1725	0.09	5.19	*p* < 0.001	*p* < 0.001
	INS.R, OFL.R	16 36 08	450	−0.1	−5.24	*p* < 0.001	*p* < 0.001
MPFC	SMA.R, SMA.L SFG.R, ACC, PCC	00 10 74	8313	−0.1	−9.05	*p* < 0.001	*p* < 0.001
	Thalamus, HIP.L	02 32 16	320	−0.1	−5.15	*p* < 0.001	*p* < 0.001

*Peak significance (FWE-corrected, threshold of *p* = 0.05)*.

#### Group Differences in MPFC Connectivity

The SVCI group exhibited hypoconnectivity between the MPFC and the bilateral supplementary motor area (SMA), thalamus, the SFG.L, ACC, superior parietal lobe (SPL), and hippocampus (HIP) when compared with the controls (Table 2; Figure 1).

### Comparison of GM Volume between SVCI Patients and the Controls

Compared with the controls, SVCI patients exhibited widespread cortical GM volume loss in both the cortical and the subcortical brain regions (Table 3). The cortical regions included several frontal regions (i.e., the OFL), and temporal regions [i.e., the bilateral MTG and left ITG, the STG, and the parahippocampal gyrus (PHG)] regions, one occipital region [the left middle occipital gyrus (MOG)], and one parietal region (the PCC). The subcortical regions included the bilateral thalamus, the INS, and the amygdala.

**Table 3:** **VBM analysis showing GM volume reduction in SVCI**.

ID	Region	Side (L/R)	Size (voxels)	Peak MNI coordinates (mm)			*t*-value
				*x*	*y*	*z*
1	Frontal mid orb lobe	R	1685	33.0	−46.5	51.0	7.029
2	Frontal mid orb gyrus	R	719	21.0	−58.5	60.0	7.106
3	Thalamus	R	436	61.5	−22.5	−3.0	7.896
4	Thalamus	L	371	−27.0	3.0	−1.5	5.523
5	Middle occipital gyrus	L	263	−28.5	−94.5	4.5	5.745
6	Frontal sup orb	L	256	−33.0	−1.5	3.0	6.736
7	Posterior cingulate	L	189	−43.5	−88.5	−4.5	7.953
8	Parahippocampal gyrus	L	139	−27.0	−19.5	72.0	4.933
9	Inferior temporal gyrus	L	116	−51.0	−3.0	−39.0	5.892
10	Middle temporal gyrus	L	111	−54.0	10.5	−27.0	5.602
11	Middle temporal gyrus	R	110	19.5	−49.5	4.5	4.119
12	Insula	L	110	−36.0	−21.0	12.0	4.347
13	Superior temporal gyrus	L	106	−4.5	45.0	15.0	4.533
14	Posterior cingulate	R	89	45.0	−49.5	48.0	5.353
15	Superior temporal gyrus	L	79	−52.5	15.0	−15.0	5.099
16	Amygdala	R	78	27.0	−34.5	−4.5	4.184

*L/R, left/right hemisphere; MNI, Montreal Neurological Institute. Voxel resolution = 1.5 mm × 1.5 mm × 1.5 mm. The regions listed were defined according to the AAL atlas provided by SPM*.

### Relationship between the Altered Functional Connectivity and the Results of Neuropsychological Tests in SVCI Patients

**Figure 2:**
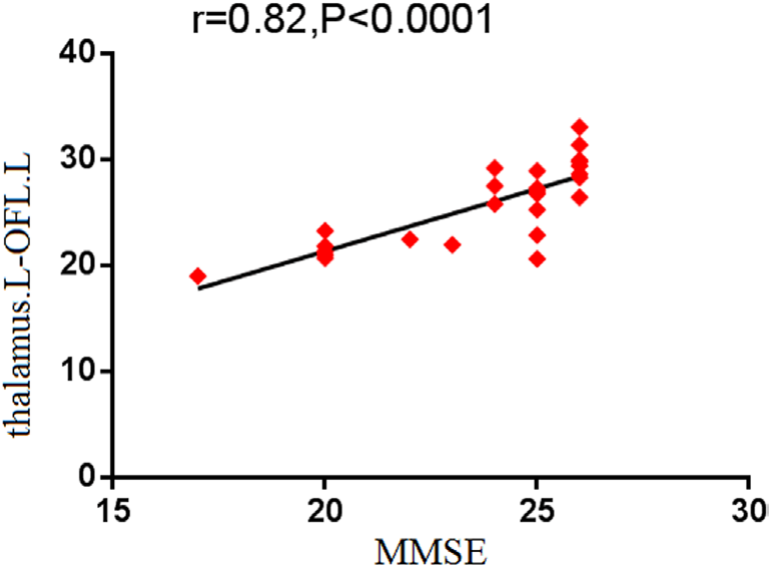
**Correlation results between altered functional connectivity and neuropsychological tests scores**. Pearson correlation analyses reveal that MMSE scores positively correlated with the functional connectivity between the left thalamus and the left OFL, which showed deceased functional connectivity in SVCI (*p* < 0.05, FDR-corrected). OFL, orbitofrontal lobe.

To identify the relationship between the strength of functional connectivity and the clinical scores in SVCI, the average functional connectivity strength of all voxels in each of the above regions was extracted separately. We detected that the MMSE performance was positively correlated with the functional connectivity between the left thalamus and the left OFL (*r* = 0.82, *p* < 0.001; Figure 2).

## Discussion

Recent studies have shown disrupted resting-state functional connectivity between the PCC and several other brain regions in SVCI. This study provided new evidence for the disconnection syndrome by investigating the connectivity between the thalamus and MPFC and all other brain regions. Three major findings were revealed. First, both the thalamus connectivity and the MPFC connectivity with a set of brain regions were disrupted in SVCI, whereas the functional connectivity between the thalamus and several parts of the frontal regions were increased. Second, the structures with reduced volume mostly coincided with the regions with aberrant functional connectivity except for the occipital region. Third, the cognitive performance was closely correlated with functional connectivity between the left thalamus and the left OFL in SVCI. To the best of our knowledge, this is the first study to explore the thalamus and the MPFC functional connectivity in relation to the cognition in SVCI.

Previous studies identified both the thalamus and the MPFC as vital nodes in the executive network (Zhang et al., 2008; Unschuld et al., 2013), and as important regions in the DMN. The thalamus is a critical structure involved in the frontal-subcortical circuits and plays a vital role in cognitive functions, such as execution, attention, and memory. When we focused on the thalamus as a seed, we found that several frontal lobe regions (the OFL and SFG) appeared to have disrupted connectivity with the thalamus in SVCI. The relationship between the OFL and executive dysfunction has been well established and is supported by the fact that the patients with orbito-basal lesions in traumatic brain injury always exhibit executive dysfunction (de Guise et al., 2015). Additionally, the OFL is one of the origins of frontal-subcortical circuits and has reciprocal connections with the thalamus, which provides the direct structural basis for functional connectivity (Bonelli and Cummings, 2007). Given the importance of the OFL in frontal-subcortical circuits, these results may explain several of the symptoms exhibited in SVCI, such as impaired executive function and decreased attention, and provide further evidence that disrupted thalamo-frontal functional connectivity patterns may underlie the impaired cognitive ability of SVCI patients. More importantly, in our study, the strength of functional connectivity between the thalamus and the OFL was positively correlated with the MMSE, which indicated that cognitive ability is closely correlated with the functional connectivity index of this region. In the temporal lobe regions, we found that both the FFG and the STG showed disrupted connectivity with the thalamus. According to a previous study (Apicella et al., 2013), the FFG is regarded as an important brain structure in memory as well as in processing facial features. The STG is a key structure in processing social stimuli for self-regulation of social behavior (Adolphs, 2003). This finding of disrupted connectivity of the FFG and STG with the thalamus may contribute to the impairment of memory and the decline in processing in SVCI. We also noticed that reduced functional connectivity with the thalamus existed in the subcortical regions (the bilateral INS and PUT). The INS is involved in diverse functions, such as somatosensation, interoception, motivation, and the maintenance of homeostasis (Boccardi et al., 2005). A combined fMRI and transcranial magnetic stimulation (TMS) study (Bestmann et al., 2004) also uncovered important functional connectivity between the INS and the thalamus. The decreased connectivity between the thalamus and the INS may be indicative of an impaired pathway between them, which may ultimately influence normal functions, including auditory processing, somatic sensation, and movement in SVCI patients. Our findings provide new evidence that functional connectivity between the INS and the thalamus is of considerable importance. The PUT (de Jong et al., 2008), another important subcortical nucleus, is known to participate in many different neuronal pathways, referring to regulation of cognition and behavior, including memory and mood, which are impaired in SVCI. Taken together, these findings highlighted a key role for subcortical regions in the neurodevelopment of SVCI.

Apart from the disrupted functional connectivity, we also observed that the right regions of the IFG and MFG showed increased connectivity with the thalamus. Our result was in concordance with a previous study (Li et al., 2012) that showed distinctly increased activation in the IFG and MFG during tasks in individuals with subcortical ischemic VCIND (SIVCIND) in comparison with the controls. Both the IFG and the MFG regions are involved in the executive/attention network (Eliasova et al., 2014; Japee et al., 2015). It has been reported that the brain has a buffer or reserve capacity that enables it to resiliently face the pathological attacks caused by aging or disease (Staff, 2012). Thus, the enhanced functional interaction with the thalamus may be considered as a compensatory reallocation of the decreased functional connectivity or as adaptation to limit executive/attention dysfunction as the disease progressed.

Similar to the thalamus, the MPFC is critically involved in numerous cognitive functions, and particularly executive control and attention as well as working memory, which are the functions most frequently impaired in SVCI. Through its heavy projection to or from cortical and subcortical areas, the MPFC may also act as a central hub in the brain circuitry for the integration and projection of information from numerous input and output structures (Riga et al., 2014). In the present study, we observed hypoconnectivity between the MPFC and the SMA, SFG, and ACC. According to a previous study, the SMA, as the origin region of the motor circuit, plays an essential part in motor functions (Bonelli and Cummings, 2007). This finding suggests that the disruption of connectivity is extended to the motor cortex in SVCI, which results in deficits of motor function, such as gait dysfunction and slowness. Both the SFG and the ACC are known to be involved in a variety of cognitive and other diverse functions, such as emotion, language, action, and execution/attention (Li et al., 2013; Minzenberg et al., 2015). Thus, the disruption of the functional connectivity between the MPFC and the SFG or ACC in SVCI may further underlie the impaired executive function of SVCI patients. We also noted that certain memory-related regions experienced disruption in functional connectivity. For example, functional connectivity was disrupted between the MTG/STG and the thalamus as well as between the MPFC and the HIP. It is well established that the MTG, STG, and HIP are involved in memory (Vandenberghe et al., 1996; Raettig and Kotz, 2008). Although memory dysfunction is an essential characteristic of AD, it has also been identified in SVCI patients, which may be attributable to degeneration or ischemia (Frisoni et al., 2002). Our findings, therefore, provide new evidence for memory dysfunction in SVCI. We also observed that the thalamus presented decreased connectivity with the MPFC, underscoring the important role of the thalamus in frontal-subcortical circuits.

Moreover, to clarify the relationship between functional and structural alterations, we examined the GM volume in SVCI. VBM results demonstrated that, relative to controls, SVCI patients exhibited significant cortical thinning in the frontal, temporal, parietal, and occipital regions as well as in certain subcortical areas, including the bilateral thalamus, INS, and PUT. These observations are congruent with previous findings (Seo et al., 2010; Yi et al., 2012). We also noted that in SVCI structures with reduced volume mostly coincided with the regions with aberrant functional connectivity, except for the occipital region without functional alteration. Occipital region atrophy has been previously reported in patients with SVMCI (Yi et al., 2012). These inconsistent findings regarding functional and structural alterations suggest that gray volume reduction in these functional brain areas might be a cause of functional deficits in SVCI. Taken together, the functional and structural abnormalities in these brain areas are suggestive of a biological basis for cognitive deficits in SVCI.

There are several limitations of our study. First, we cannot completely exclude the influence of drugs on brain activity. Second, similar to most resting-state fMRI studies, the interference of certain potential confounding factors, such as respiratory and cardiac cycle artifacts, slow sampling rate could not be excluded, despite band-pass filtering of 0.01–0.08 Hz. Third, because of the small sample size, the difference in functional connectivity between SVCI patients with mild and severe cognitive impairment levels could not be assessed adequately. To better understand the development of SVCI, studies with larger samples will be required to investigate the functional attribution of SVMCI and subcortical VD (SVD) patient groups separately.

## Conclusion

In summary, the current study examined bilateral thalamus and MPFC connectivity with all the other brain regions and morphological alterations using the resting fMRI and VBM. We found that the functional connectivity of the thalamus and MPFC was decreased in both cortical and subcortical regions, which also exhibited significant atrophy in structure, whereas the increased functional connectivity was targeted in the frontal lobe. Moreover, the functional connectivity between the left thalamus and the left OFL was correlated with the cognitive performance. These findings highlight the importance of the thalamus and the MPFC in frontal-subcortical circuits and improve our understanding of the mechanism of cognition dysfunction in SVCI patients.

## Author Contributions

Conceived and designed the experiments: XZ, XH, ZS, and YY. Performed the experiments: XZ, XH, HW, LX, and XZ. Analyzed the data: XZ, XH, and CZ. Contributed reagents/materials/analysis tools: XZ and XH. Wrote the paper: XZ. Figures processing: XZ, XH, and CZ.

## Conflict of Interest Statement

The authors declare that the research was conducted in the absence of any commercial or financial relationships that could be construed as a potential conflict of interest.

## Footnotes

^1^http://www.fil.ion.ucl.ac.uk/spm

^2^http://www.ansir.wfubmc.edu
